# Comparison of fully covered self-expandable metal biliary stents with or without anchoring double-pigtail plastic stents: a systematic review and meta-analysis

**DOI:** 10.1016/j.igie.2025.12.002

**Published:** 2025-12-09

**Authors:** Samuel Tanner, Fadi Hawa, Alejandra Vargas, Priyata Dutta, Antonio Facciorusso, Janice Lester, Jean M. Chalhoub, Jorge D. Machicado

**Affiliations:** 1 Division of Gastroenterology and Hepatology, University of Michigan, Ann Arbor, Michigan, USA; 2 Division of Gastroenterology and Hepatology, Ohio State University, Columbus, Ohio, USA; 3 Department of Internal Medicine, Eastern Virginia Medical School, Norfolk, Virginia, USA; 4 Department of Internal Medicine, Trinity Health, Ann Arbor, Michigan, USA; 5 Gastroenterology Unit, Department of Experimental Medicine, Università del Salento, Lecce, Italy; 6 Health Sciences Library, North Shore-LIJ Health System, Great Neck, New York, USA; 7 Division of Gastroenterology and Hepatology, Staten Island University Hospital, Staten Island, New York, USA

## Abstract

**Background and Aims:**

Fully covered self-expandable metal stents (FcSEMSs) have become the mainstay of treatment for a variety of biliary pathologies. However, FcSEMSs have been associated with a greater rate of stent migration than other types of stents. A technique of anchoring double-pigtail plastic stents (DPPSs) has been proposed to minimize migration of biliary FcSEMSs. Herein, we evaluated the efficacy of anchoring DPPSs among patients treated with biliary FcSEMSs.

**Methods:**

We performed a systematic review using PubMed, EMBASE, and Web of Science from database inception through August 2023. Full-text articles comparing FcSEMSs with or without anchoring DPPSs were included. We evaluated the following outcomes: (1) stent migration; (2) stent occlusion; (3) duration of stent patency; (4) cholangitis; (5) unplanned reinterventions; and (6) adverse events. Meta-analysis was carried out using random-effect models and reported as odds ratios (ORs) with corresponding 95% confidence intervals (95% CIs).

**Results:**

Four studies encompassing 489 patients were included. Patients with anchoring DPPSs had a 67% reduction in FcSEMS migration compared with those without anchoring DPPSs (OR: 0.33; 95% CI, 0.19-0.57; *I*^2^ 0%). Anchoring DPPSs increased the mean duration of FcSEMS patency by 83 days compared with FcSEMSs alone (95% CI, 46-120; *I*^2^ 94%). No differences in other clinical end points or adverse events were observed.

**Conclusions:**

Anchoring DPPSs reduced the risk of FcSEMS migration and increased the duration of FcSEMS patency without an increased risk of adverse events. Future studies are needed to corroborate these findings, determine the optimal DPPS technique, and to compare DPPS placement with other antimigratory modalities.

## Introduction

Fully covered self-expandable metal stents (FcSEMSs) are recommended by recent American Society for Gastrointestinal Endoscopy guidelines over plastic stents and uncovered metal stents for the management of distal benign or malignant biliary strictures.^1^, ^2^, ^3^, ^4^ The use of FcSEMSs also plays an important role in the management of postsphincterotomy bleeding and bile leaks.^5^, ^6^, ^7^ Advantages of an FcSEMS include its larger diameter than plastic stents and its lower risk of tissue hyperplasia and tumor ingrowth than uncovered metal stents.^8^^,^^9^ However, stent migration is a common adverse event of FcSEMSs, with estimates ranging from 11% to 54%.^10^^,^^11^ Stent migration can result in stent failure, cholangitis, hospitalization, or unplanned endoscopic retrograde cholangiopancreatography (ERCP).

To reduce the risk of FcSEMS migration, different antimigratory techniques have been described. These techniques can be either intrinsic (eg, addition of anchoring fins, use of a partially covered stent, and increased radial force) or extrinsic (eg, use of anchoring double-pigtail plastic stents [DPPSs], securing the distal retrieval loop via clipping).^12^, ^13^, ^14^ Intrinsic techniques may be beneficial, but have been associated with adverse outcomes, including bleeding and ulceration from anchoring flaps^15^ or tissue ingrowth involving the uncovered portion of metal stents, making stent retrieval difficult.^16^^,^^17^ To reduce the issues with intrinsic antimigratory properties, prior studies have reported the use of anchoring DPPSs. In this technique, the proximal tip of the plastic stent is placed above the FcSEMS, ideally in the intrahepatic bile ducts if technically feasible, to minimize FcSEMS migration. The plastic stent can be placed either coaxially to (within) the FcSEMS or in a side-by-side configuration.^18^^,^^19^

Prior studies evaluating the efficacy of placing DPPSs to prevent FcSEMS migration have reported conflicting results.^18^, ^19^, ^20^, ^21^, ^22^ These studies vary in design, technique, and indications for FcSEMS placement. Therefore, we conducted a systematic review and meta-analysis that aimed to better estimate the efficacy and safety of anchoring DPPSs in reducing the risk of FcSEMS migration.

## Methods

### Search strategy

A comprehensive literature search was designed and executed by an experienced health science librarian (J.L.) using PubMed, EMBASE, and Web of Science, from the inception of each database through August 2023. A combination of Medical Subject Headings terms was used, including (1) “bile duct,” (2) “ERCP,” (3) “fully covered metal stent,” and (4) “double-pigtail plastic stent.” The complete search strategies are available in Supplementary Appendix 1, available online at www.igiejournal.org. This study was conducted according to the Preferred Reporting Items for Systematic reviews and Meta-Analyses statement.^23^

### Eligibility criteria

To be eligible, studies were required to be fully published randomized controlled trials (RCTs), observational studies, or case series with more than 10 patients comparing FcSEMSs with or without anchoring DPPSs. Studies that included coaxial and side-by-side placement of DPPSs were eligible. Studies that were noncomparative, published as abstract alone, or not in English were excluded. Case reports, editorials, review articles, letters to the editor, guidelines, and meta-analyses were also excluded.

### Outcome measures

The primary outcome was the rate of the FcSEMS migration. This was defined as the radiological or endoscopic evidence of proximal or distal stent movement away from its initial position at the time of placement. Secondary outcomes included (1) stent occlusion by stone or sludge or by tumor; (2) duration of stent patency; (3) cholangitis; (4) unplanned reinterventions; and (5) adverse events, including pancreatitis, cholecystitis, bleeding, and perforation. Stent patency was defined as the time between stent placement and stent revision for any stent dysfunction attributable to migration, occlusion, or other causes requiring reintervention. Clinical success was also evaluated for patients treated for biliary stricture and defined as the reduction in total bilirubin levels to less than half of the pretreatment levels within 4 weeks of placement.

### Data extraction and quality assessment

Two investigators (F.H. and A.V.) independently screened the titles and abstracts and then reviewed relevant manuscripts. Any disagreements were resolved through consensus with the corresponding author (J.D.M.). After selection of eligible studies, 2 investigators (F.H. and A.V.) independently abstracted data and assessed risk of bias using the Newcastle-Ottawa Scale (NOS).^24^ Any disagreements were resolved by discussion with the corresponding author (J.D.M.).

### Statistical analysis

Categorical outcome measures are summarized as pooled proportions or mean difference with 95% confidence intervals (CIs). The primary and secondary outcomes were compared across the 2 groups (with or without anchoring DPPSs) using odds ratios (ORs). Heterogeneity was estimated using the *I*^2^ statistic. Because between-study heterogeneity was expected, the pooled estimates were computed using the random-effects models and the DerSimonian and Laird method. A *P* value of < .05 was considered statistically significant. Statistical analysis was performed using Review Manager (The Cochrane Collaboration, Oxford, UK) and the R metafor package for the Jamovi statistical software (Sydney, Australia).

## Results

### Study selection and quality assessment

**Figure 1:**
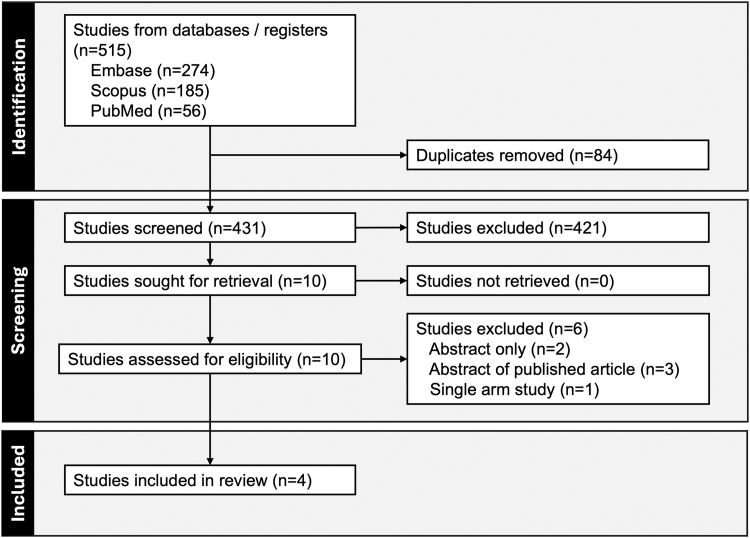
Systematic review flow diagram.

**Supplementary Figure 1:**
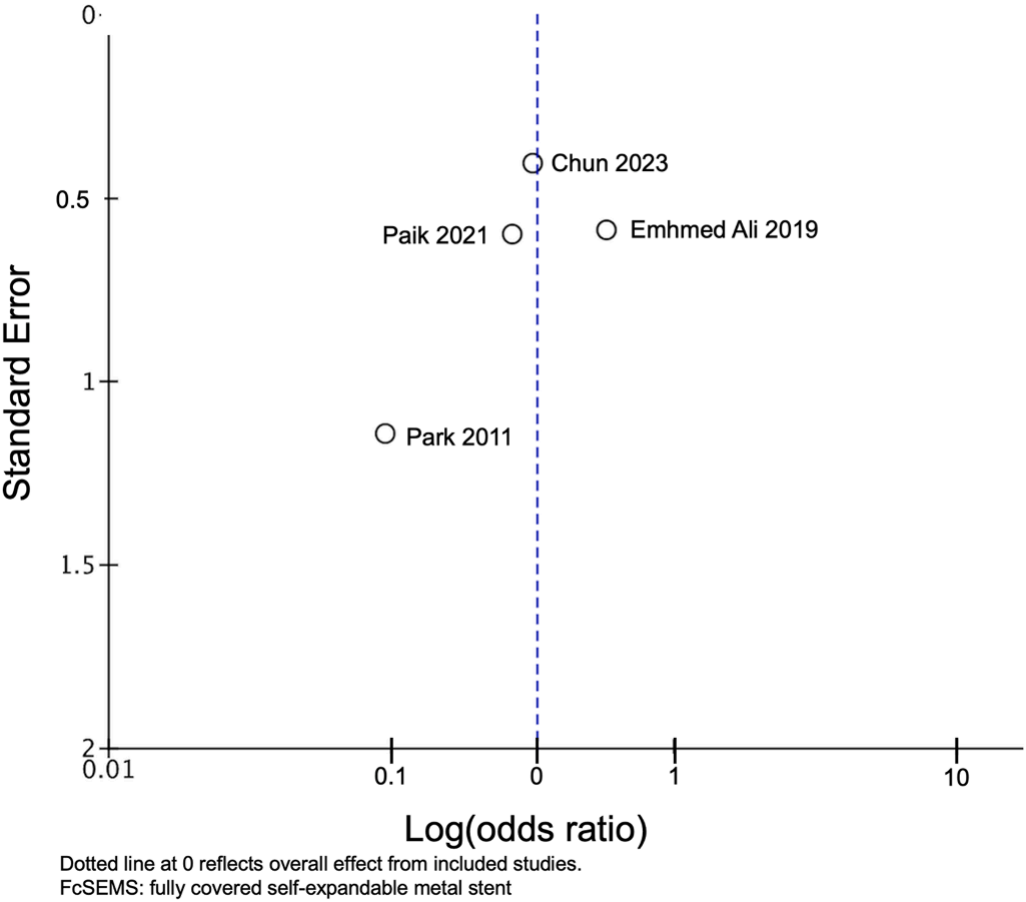
Funnel plot of studies comparing migration risk between anchored and nonanchored FcSEMS.

The initial search yielded 515 studies, of which 84 were duplicates. A total of 431 titles were screened, and subsequently 10 full-text articles were reviewed (Fig. 1). We identified 4 publications encompassing 489 patients that fulfilled eligibility criteria. This included 2 RCTs^19^^,^^21^ and 2 retrospective studies^18^^,^^22^ published between 2011 and 2023. All studies were rated as high quality (NOS ≥6, Supplementary Table 1, available online at www.igiejournal.org). Visual inspection of the inverted funnel plots did not suggest publication bias (Supplementary Fig. 1, available online at www.igiejournal.org).

### Study characteristics

Table 1 describes the baseline and procedure characteristics of the studies included in the systematic review. A total of 489 patients (59% male, median age 65 years [interquartile range = 34.5-89]) underwent FcSEMSs placement. Of the patients, 234 underwent placement of FcSEMSs with anchoring DPPSs, whereas the remaining 255 underwent placement of FcSEMSs alone. Among the 4 studies included, 2 studies evaluated DPPSs for FcSEMSs in the setting of malignant biliary stricture,^18^^,^^19^ 1 study evaluated DPPSs for FcSEMSs in the setting of benign biliary strictures,^21^ and 1 study evaluated DPPSs for FcSEMSs in the setting of any indication of FcSEMS placement.^22^ The majority of patients had an FcSEMS placed for biliary strictures of malignant (71%) or benign etiology (24%), while 5% had it for bile leak, cholangitis, or postsphincterotomy bleeding. Among patients with biliary strictures, most strictures were located in the distal common bile duct (97%) and had a median length of 17.6 mm [interquartile range = 11-65]. The follow-up period after FcSEMS placement ranged from 6 to 12 months.

**Table 1:** Baseline and procedure characteristics of the studies included in the systematic review

First author	Year	Country	Design	Number of centers	Number of patients		Indication for biliary drainage			Anchoring method	Median follow-up (months)
					Nonanchored	Anchored	Benign strictures	Malignant strictures	Other		
Park 2011^21^	2011	South Korea	RCT	1	17	16	33	0	0	Coaxial	6
Emhmed Ali 2019^22^	2019	United States	RS	1	138	65	82	95	26∗	Coaxial	NR
Paik 2021^19^	2021	South Korea	RCT	4	35	33	0	68	0	Coaxial	12
Chun 2023^18^	2023	South Korea	RS	2	65	120	0	185	0	Side-to-side	7

*NR*, Not reported; *RCT*, randomized controlled trial; *RS*, retrospective.

∗ On the basis of the reported data, a total of 82 patients had benign strictures, and 95 patients had malignant strictures. Therefore, 26 patients underwent fully covered self-expandable metal stent placement for a nonstricture-related pathology such as bile leak or postsphincterotomy bleeding.

Stent configurations are reported in Supplementary Table 2, available online at www.igiejournal.org. The diameter of the self-expandable metal stent ranged from 8 to 10 mm, and the length ranged from 4 to 10 cm. Six different brands of FcSEMSs were used, including (n = 33, S&G Biotech, Seongnam, South Korea), VIABIL (n = 153, Gore Medical, Flagstaff, Ariz, USA), WallFlex (n = 50, Boston Scientific, Marlborough, Mass, USA), ARISTENT (n = 165, Daewoong, Seoul, South Korea), and BONASTENT (n = 88, Standard Sci-Tech, Seoul, South Korea). These stents have a variety of intrinsic antimigratory properties, including distal and proximal flares, although only 1 brand of FcSEMSs had antimigratory fins (VIABIL). Three studies reported on the type of DPPS used, all of which were the 5F or 7F Zimmon Biliary Stent (Cook Medical, Bloomington, Ind, USA).^18^^,^^19^^,^^21^ The 1 study that did not report the brand of DPPS used a 7F or 10F DPPS.^22^ DPPSs were placed coaxially within the FcSEMS in 3 studies^19^^,^^21^^,^^22^ or in a side-to-side configuration to the FcSEMS in 1 study.^18^ Technical success of anchoring DPPS placement was 100% among the included studies.

### Primary outcome

**Figure 2:**
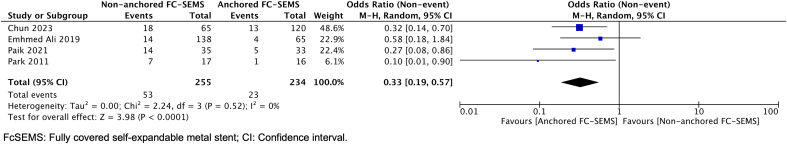
Forest plot comparing migration rate between anchored and nonanchored FcSEMS.

All studies (n = 489) reported the primary outcome. Migration of FcSEMSs occurred in 53 of 255 patients with nonanchored FcSEMSs and in 23 of 234 in those with anchored FcSEMSs. The use of anchoring DPPSs was associated with a 67% reduction in the risk of FcSEMS migration than in those without anchoring plastic stents (OR: 0.33; 95% CI, 0.19-0.57; *P* < .001; *I*^2^ = 0%) (Fig. 2).

### Secondary outcomes

**Figure 3:**
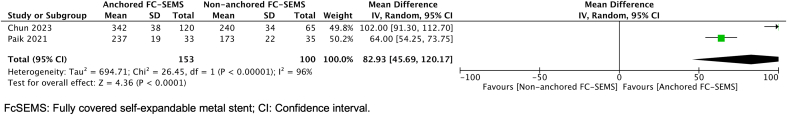
Forest plot comparing the duration of stent patency between anchored and nonanchored FcSEMS.

**Supplementary Figure 2:**
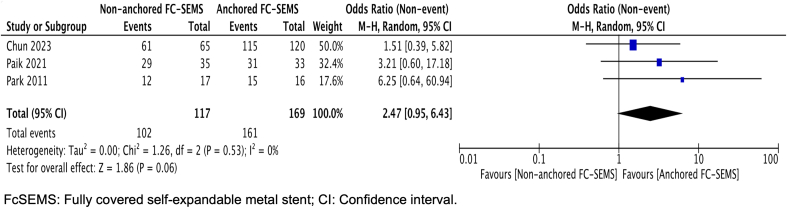
Forest plot of studies comparing clinical success between anchored and nonanchored FcSEMS.

**Supplementary Figure 3:**
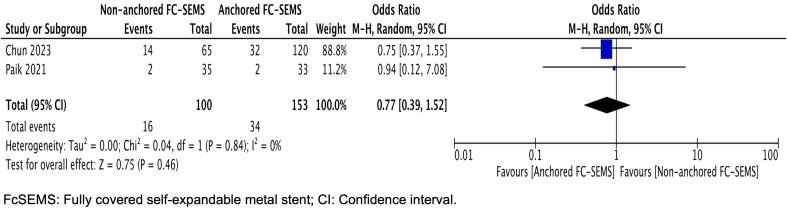
Forest plot comparing the rate of occlusion between anchored and nonanchored FcSEMS.

**Supplementary Figure 4:**
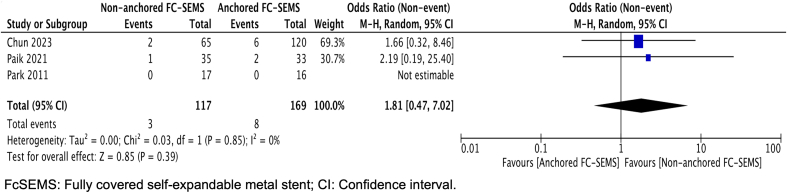
Forest plot comparing the risk of cholangitis between anchored and nonanchored FcSEMS.

**Supplementary Figures 5:**
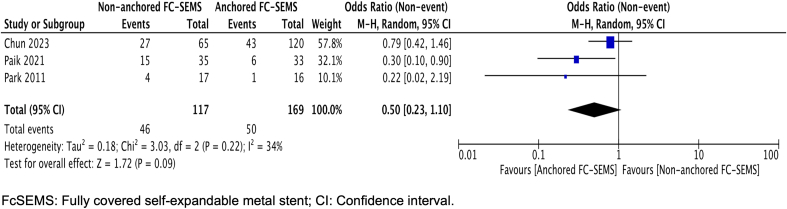
Forest plot comparing the need for unplanned interventions after stent placement between anchored and nonanchored FcSEMS.

Secondary outcomes are described in Table 2 and Supplementary Figure 2, Supplementary Figure 3, Supplementary Figure 4, Supplementary Figures 5, available online at www.igiejournal.org. Two studies in which the stent was left as destination therapy for distal malignant biliary obstruction (n = 253) reported stent patency. Among these patients, those who underwent FcSEMS placement with anchoring DPPSs had 83-day-longer stent patency than with an FcSEMS alone (95% CI, 46-120 days; *P* < .001; *I*^2^ = 94%) (Fig. 3). There was a nonsignificant trend toward fewer unplanned reinterventions required with FcSEMSs when a DPPS was inserted (OR: 0.50; 95% CI, 0.23-1.10; *P* = .09; *I*^2^ = 34%). No significant differences were noted between the DPPS and non-DPPS groups in terms of clinical success, stent occlusion, or cholangitis.

**Table 2:** Outcomes of FcSEMS placement with or without anchoring DPPSs

Study outcomes	Number of studies	Anchored FcSEMS	Nonanchored FcSEMS	OR or MD (95% CI)	*P* value	Heterogeneity (*I*^2^)
Stent migration	4	23/234	53/255	0.33 (0.19-0.57)	<.001	0%
Duration of stent patency (days)	2	153	100	83 (46-120)∗	<.001	96%
Clinical success	3	161/169	102/117	2.47 (0.95-6.43)	.06	0%
Stent occlusion	2	34/153	16/100	0.77 (0.39-1.52)	.46	0%
Unplanned interventions	3	50/169	46/117	0.50 (0.23-1.10)	.09	34%
Cholangitis	3	8/169	3/117	1.81 (0.47-7.02)	.39	0%

*CI*, Confidence interval; *DPPS*, double-pigtail plastic stent; *FcSEMS*, fully covered self-expandable metal stent; *MD*, mean difference; *OR*, odds ratio.

∗ Mean difference.

### Adverse events

**Supplementary Figure 6:**
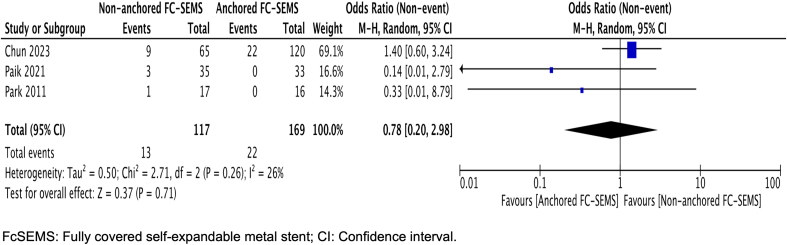
Forest plot comparing total adverse events between anchored and nonanchored FcSEMS.

Three studies (n = 286) reported adverse events (AEs) associated with FcSEMS placement with or without anchoring DPPSs (Table 3).^18^^,^^19^^,^^21^ No statistically significant difference in AEs was noted between the 2 groups (Supplementary Fig. 6, available online at www.igiejournal.org).

**Table 3:** Adverse events associated with FcSEMS placement with or without anchoring DPPSs

	Anchored FcSEMS (n = 169)	Nonanchored FcSEMS (n = 117)	OR (95% CI)	*P* value	Heterogeneity (*I*^2^)
Adverse events	22	13	0.78 (0.20-2.98)	.71	26%
Pancreatitis	8	7	–	–	–
Cholecystitis	8	5	–	–	–
Bleeding	5	1	–	–	–
Perforation	1	0	–	–	–

*CI*, Confidence interval; *DPPS*, double-pigtail plastic stent; *FcSEMS*, fully covered self-expandable metal stent; *OR*, odds ratio.

The dashes reflect that these values were not calculated due to the low number of events.

## Discussion

In this systematic review and meta-analysis, we demonstrate that anchoring FcSEMSs with DPPSs was associated with a 67% risk reduction of FcSEMS stent migration than patients without anchoring DPPSs. Furthermore, anchoring DPPSs prolonged the duration of stent patency in patients with distal malignant biliary strictures where an FcSEMS was placed as destination therapy. We also found that the placement of anchoring DPPSs was safe and with high technical success.

The antimigratory effect of the anchoring DPPS was demonstrated by each of the included studies, except in a retrospective study of 203 patients (6% in the anchoring group vs 10% in the nonanchoring group; *P* = .35).^22^ This could be attributed to multiple factors, including (1) the lack of provided procedural details on whether intrahepatic anchorage of the DPPSs proximal tip was attempted or successful, (2) an unknown follow-up period with a relatively low overall stent migration rate of 9.7%, and (3) the variety of biliary stents being used, including 1 with anchoring fins in 75% of patients. Not only did a DPPS reduce FcSEMS migration, but it was also found to be as safe as the current standard of care without a DPPS. Although none of the studies have compared DPPSs with other antimigratory mechanisms, these modalities carry risks of mucosal injury with anchoring fins, tissue ingrowth of partially covered stents making stent retrieval difficult, limited availability of novel stents, and the risk of pancreatitis because of clipping adjacent to the papilla.^12^, ^13^, ^14^^,^^25^^,^^26^ One disadvantage of placing a DPPS is the incremental time and costs required by putting an additional stent. One of the studies included in our systematic review found that this technique required 6 additional minutes.^18^ However, this additional time may not be clinically significant when considering total procedural time and the added benefits.

An important clinical finding was that the duration of FcSEMS patency was significantly increased by approximately 3 months when a DPPS was inserted. This was assessed in 2 studies that used FcSEMSs as destination therapy in patients with distal malignant biliary stricture. One study included only patients with unresectable tumors,^19^ and the other included malignancies at different stages, with 27 of 185 patients who had subsequent surgical resection.^18^ A longer duration of stent patency may decrease chemotherapy interruptions in patients receiving neoadjuvant or palliative chemotherapy regimens. It may also improve the quality of life of patients with unresectable disease, allowing them to focus on comfort and palliative interventions. Although not statistically significant, there was a trend toward a reduction of reinterventions with DPPSs, which would be expected with longer stent patency. This finding was not significant, likely because of the small sample size (n = 286) and limited power.

Two techniques for placing the anchoring DPPS in relation to the FcSEMS have been reported. Coaxial placement of the plastic stent within the FcSEMS was used in 3 studies.^19^^,^^21^^,^^22^ This method may result in en bloc migration of both the FcSEMS and DPPS if migration occurs, leading to immediate stent failure and the need for reinterventions to maintain biliary drainage. The other technique involves placing the anchoring DPPS side-by-side with the FcSEMS and was described in 1 study.^18^ This alternative technique can theoretically increase the chances of the DPPS remaining in place and ensuring biliary drainage despite the migration of the FcSEMS. Both techniques have been shown to reduce the risk of FcSEMS migration and increase stent patency in individual studies. However, a dedicated subgroup analysis by technique was not possible because only 1 study evaluated the side-by-side technique.

Our study has some limitations. Only 4 studies met the eligibility criteria, including <500 patients. This may have led to low power to detect a difference in some of the secondary end points such as reinterventions, stent occlusion, or cholangitis. However, this sample size was sufficient to find a significant difference in the primary end point (stent migration) with no heterogeneity of results. To increase the sample size, we included observational studies, which inevitably introduced selection, misclassification, and other types of bias. Nonetheless, all studies were judged as high quality using the NOS. There was heterogeneity in the interventions used across studies, with different configurations of FcSEMS and DPPS techniques. Subgroup analysis based on these intervention details was not possible because of differences among the individual studies. Although the indication for FcSEMSs was variable between studies, our study is representative of patients with distal biliary strictures (>90% of the study population), and our results are not generalizable to other populations, such as patients undergoing FcSEMS placement for short (<3-month) periods (eg, bile leak, sphincterotomy bleeding).

In conclusion, this meta-analysis showed that placement of anchoring DPPSs reduced the risk of biliary FcSEMS migration and increased the duration of biliary FcSEMS patency. This technique holds promise for patients who receive a biliary FcSEMS for malignant distal biliary stricture before neoadjuvant chemotherapy or for palliative reasons. Future RCTs powered for meaningful clinical end points are needed to confirm the benefits of DPPSs when an FcSEMS is used. Additional studies are needed to assess the role of anchoring a DPPS when using an FcSEMS in benign indications, determine the optimal DPPS technique to use, and compare DPPS placement with other antimigratory techniques.

## Patient Consent

This is a meta-analysis, so patient consent is not applicable.

## Disclosure

The following author disclosed financial relationships: J. D. Machicado: Research support from the ACG Junior Faculty Career Development Award and Robert A. Winn Excellence in Clinical Trials Career Development Award; advisory board of Amgen LCM and Pulse Biosciences. All other authors disclosed no financial relationships.

## Figures and Tables

**Supplementary Table 1:** Quality assessment of included studies (Newcastle-Ottawa Scale)

Author, years	Selection bias (out of 4)				Comparability bias (out of 2)	Outcome bias (out of 3)			Total (out of 9)
	Representativeness of exposed cohort	Selection of nonexposed cohort	Ascertainment of exposure	Baseline diagnosis		Assessment methods	Follow-up duration	Adequacy of follow-up	
Park, 2011^21^	1	1	1	1	0	1	1	1	7
Emhmed Ali, 2019^22^	1	1	1	1	0	1	0	1	6
Paik, 2021^19^	1	1	1	1	2	1	1	1	9
Chun, 2023^18^	1	1	1	1	2	1	1	1	9

Each study is judged on 3 broad perspectives (selection of study groups, comparability of the groups, and ascertainment of the outcome of interest). A study can be awarded a maximum of 1 for each item within the selection and outcome categories. A maximum of 2 can be given for comparability. A total score of Newcastle-Ottawa Scale ≥6 was defined as high quality.

**Supplementary Table 2:** FcSEMS characteristics across included studies

Author, year	FcSEMS type	Number of patients	Antimigration features	Composition
Park, 2011^21^	S&G Biotech (Seongnam, South Korea)	33	Flared ends	Nitinol wire with silicone-covered membrane with flared ends
Emhmed Ali, 2019^22^	VIABIL (Gore Medical, Flagstaff, Arizona, USA)	153	Anchoring fins	Nitinol wire exoskeleton with an ultrathin expandable polytetrafluoroethylene/fluorinated ethylene propylene (Peralume) covering
	WallFlex (Boston Scientific, Marlborough, Mass, USA)	50	Flared ends	Platinum-cored nitinol wire mesh with silicone-covered membrane with flared ends
Paik, 2021^19^	ARISTENT (Daewoong, Seoul, South Korea)	68	Flared ends	Nitinol hook and cross-wire structure with a silicone-covered membrane with flared ends
Chun, 2023^18^	BONASTENT (Standard Sci-Tech, Seoul, South Korea)	88	Flared ends	Nitinol hook and cross-wire structure with a polygonal mesh surface
	ARISTENT (Daewoong, Seoul, Korea)	97	Flared ends	Nitinol hook and cross-wire structure with a silicone-covered membrane with flared ends

*FcSEMS*, Fully covered self-expandable metal stent.

## Appendix 1

### Search strategy for the systematic review and meta-analysis

All databases searched were limited to the English language.

#### PUBMED

“fully covered metallic stents"[tiab:∼1] “fully covered metallic stent"[tiab:∼1] or “metal stent"[tiab:∼1] or “metal stents"[tiab:∼1] or metallic[tw] or “full covered metal stent"[tiab:∼1] or “full covered metal stents"[tiab:∼1] or SEMS[tw] or fcsem∗ or “Self Expandable Metallic Stents"[mesh] or “endoscopic biliary fully covered metallic stent"[tiab:∼1] or metal[tw]

AND

bile duct[tw] or biliary[tw] or ercp[tw] or rcp[tw] or “Bile Ducts"[Mesh] OR “Cholangiopancreatography, Endoscopic Retrograde"[Mesh] or “ERCP"[tiab:∼1]

AND.

DPPS OR “double pigtail plastic stents" [tiab:∼2] or “double pigtail plastic stent"[tiab:∼2] or pigtail∗ or coaxial[tw] or “double pigtail"[tw])

#### EMBASE

“fully near/2 covered near/2 metallic near/2 stent∗”:ti,ab,kw OR ‟metal stent∗”:ti,ab,kw OR metallic:ti,ab,kw OR ‟full near/2 covered metal near/2 stent∗”:ti,ab,kw OR sems:ti,ab,kw OR ‟self near/2 expandable near/2 metallic near/2 stent∗”:ti,ab,kw OR ‟endoscopic near/2 biliary near/2 fully near/2 covered near/2 metallic near/2 stent∗”:ti,ab,kw

dpps:ti,ab,kw OR ‟double near/2 pigtail near/2 plastic near/2 stent∗”:ti,ab,kw OR ‟double near/2 pigtail near/2 plastic near/2 stent∗”:ti,ab,kw OR pigtail∗:ti,ab,kw OR coaxial:ti,ab,kw OR ‟double near/2 pigtail”:ti,ab,kw.

‟bile duct∗”:ti,ab,kw OR biliary:ti,ab,kw OR ercp:ti,ab,kw OR rcp:ti,ab,kw OR ‟bile duct∗”:ti,ab,kw OR ‟bile duct”/exp OR ‟endoscopic retrograde cholangiopancreatography”/exp OR ‟endoscopic near/2 retrograde near/2 cholangiopancreatography”:ti,ab,kw.

#### WEB OF SCIENCE

dpps or pigtail∗

‟bile duct∗” OR biliary OR ercp OR rcp OR ‟endoscopic retrograde”

Metal∗ or sems or (self-expandable metallic stent∗)
